# Supplementary material for: Cytokine signaling converging on IL11 in ILD fibroblasts provokes aberrant epithelial differentiation signatures
Source: Front Immunol. 2023 May 17;14:1128239. doi: 10.3389/fimmu.2023.1128239 (PMC10230276; doi:10.3389/fimmu.2023.1128239)
Supplement: Supplementary file 1 [file DataSheet_1.pdf]

Supplementary Material

1 Supplementary Figures and Tables

1.1 Supplementary Tables

Supplementary Table 1. **Antibodies and dilutions for immunofluorescence staining**

**Antibody**	**Manufacturer**	**Host Species**	**Isotype**	**Reactivity**	**Clonality**	**Dilution**
ALB	R&D Systems<br>Cat# MAB1455<br>Clone# 188835	mouse	IgG2a	human, mouse, rat	monoclonal	1:50
α- SMA	Abcam<br>Cat# ab124964<br>Lot# GR303485-6	rabbit	NA	human, mouse, rat	monoclonal	1:200
CPM	Fujifilm Wako Chemicals<br>Cat# 014-27501	mouse	IgG2b	human	monoclonal	1:200
NKX2.1	Invitrogen<br>Cat# MA5-13961<br>Clone# 8G7G3/1	mouse	IgG1 κ	human, mouse, rat	monoclonal	1:50
Pro SP-C	Millipore<br>Cat # AB3786<br>Lot# 2464523	rabbit	IgG	human, mouse, rat	polyclonal	1:200
Alexa Flour 488	Invitrogen	anti-mouse	IgG1			1:500
Alexa Flour 647	Invitrogen	anti-rabbit				1:500
DAPI	Sigma					1:1000

Supplementary Table 2. **Primer sequences for qPCR**

**Gene symbol**	**Forward**	**Reverse**
ß-Actin	CAATGTGGCCGAGGACTTTG	CATTCTCCTTAGAGAGAAGTGG

BMP4	TAGCAAGAGTGCCGTCATTCC	GCGCTCAGGATACTCAAGACC
CDH2	TCAGGCGTCTGTAGAGGCTT	ATGCACATCCTTCGATAAGACTG
Col1A1	TACAGAACGGCCTCAGGTACCA	ACAGATCACGTCATCGCACAAC
HRPT	AAGAGCTATTGTAATGACCAGT	CAAAGTCTGCATTGTTTTGC
ITGB6	TCCATCTGGAGTTGGCGAAAG	TCTGTCTGCCTACACTGAGAG
KRT8	CAGAAGTCCTACAAGGTGTCCA	CTCTGGTTGACCGTAACTGCG
MMP7	GAGTGAGCTACAGTGGGAACA	CTATGACGCGGGAGTTTAACAT
SPC	CACCTGAAACGCCTTCTTATCG	TTTCTGGCTCATGTGGAGACC
VIM	GACGCCATCAACACCGAGTT	CTTTGTCGTTGGTTAGCTGGT

Supplementary Table 3. **List of upregulated proteins compared $F_{Control}$ vs. F_{ILD} identified by mass spectrometry**

**Protein FDR confidence**	**Accession**	**Unique peptides**	**Gene symbol**	**Description**	**Abundance ratio**	**Abundance ratio P-value**	**Abundance ratio adj. P-value**
High	Q68BL8	2	OLFML2B	Olfactomedin-like protein 2B	100,00	0,0000	0,0000
High	Q9C0H2	3	TTYH3	Protein tweety homolog 3	100,00	0,0000	0,0000
High	Q96FV2	2	SCRN2	Secernin-2	100,00	0,0000	0,0000
High	Q9UHX1	3	PUF60	Poly(U)-binding-splicing factor PUF60	100,00	0,0000	0,0000
High	Q16630	2	CPSF6	Cleavage and polyadenylation specificity factor subunit 6	100,00	0,0000	0,0000
High	Q00888	8	PSG4	Pregnancy-specific beta-1-glycoprotein 4	8,81	0,0000	0,0000
High	P17302	3	GJA1	Gap junction alpha-1 protein	7,85	0,0000	0,0000
High	P09341	2	CXCL1	Growth-regulated alpha protein	7,06	0,0000	0,0000
High	P19876	3	CXCL3	C-X-C motif chemokine 3	6,42	0,0000	0,0000

High	Q14956	2	GPNMB	Transmembrane glycoprotein NMB	6,02	0,0000	0,0000
High	P48307	11	TFPI2	Tissue factor pathway inhibitor 2	4,92	0,0000	0,0000
High	Q14627	3	IL13RA2	Interleukin-13 receptor subunit alpha-2	4,46	0,0000	0,0000
High	P20809	3	IL11	Interleukin-11	4,31	0,0000	0,0000
High	Q6FHJ7	2	SFRP4	Secreted frizzled-related protein 4	4,05	0,0000	0,0001
High	P10145	2	CXCL8	Interleukin-8	3,75	0,0000	0,0000

Supplementary Table 4. **List of downregulated proteins compared $F_{Control}$ vs. F_{ILD} identified by mass spectrometry**

**Protein FDR confidence**	**Accession**	**Unique peptides**	**Gene symbol**	**Description**	**Abundance ratio**	**Abundance ratio P-value**	**Abundance ratio adj. P-value**
High	Q15198	5	PDGFRL	Platelet-derived growth factor receptor-like protein	0,49	0,0025	0,0325
High	P10321	4	HLA-C	HLA class I histocompatibility antigen, C alpha chain	0,47	0,0012	0,0172
High	P22352	8	GPX3	Glutathione peroxidase 3	0,43	0,0002	0,0034
High	Q9NZP8	7	C1RL	Complement C1r subcomponent-like protein	0,42	0,0001	0,0024
High	P24821	87	TNC	Tenascin	0,42	0,0043	0,0500
High	Q9NR99	36	MXRA5	Matrix-remodeling-associated protein 5	0,42	0,0041	0,0488
High	Q13822	29	ENPP2	Ectonucleotide pyrophosphatase/phosphodiesterase family member 2	0,42	0,0041	0,0485
High	Q76M96	25	CCDC80	Coiled-coil domain-containing protein 80	0,41	0,0039	0,0473
High	O43854	20	EDIL3	EGF-like repeat and discoidin I-like domain-containing protein 3	0,41	0,0038	0,0457
High	Q9NRN5	13	OLFML3	Olfactomedin-like protein 3	0,41	0,0038	0,0458
High	P02461	61	COL3A1	Collagen alpha-1(III) chain	0,41	0,0035	0,0434

High	P62906	7	RPL10A	60S ribosomal protein L10a	0,41	0,0031	0,0391
High	P00746	15	CFD	Complement factor D	0,41	0,0031	0,0391
High	O00339	17	MATN2	Matrilin-2	0,40	0,0002	0,0039
High	O15232	11	MATN3	Matrilin-3	0,40	0,0001	0,0023

1.2 Supplementary Figures

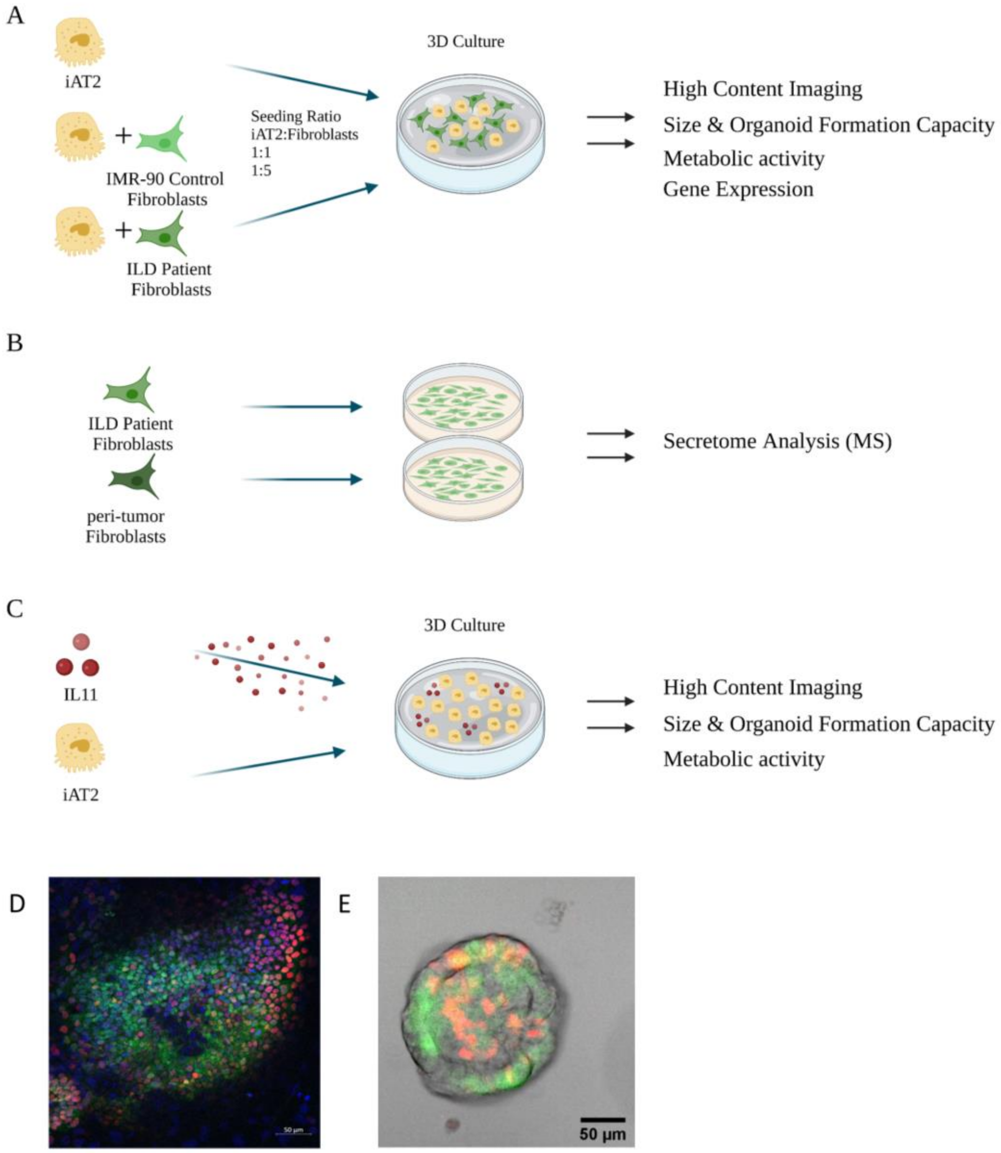

Supplementary Figure 1 (A-C) Schematic workflow of the experiments. Created with BioRender.com. (D) Immunofluorescence of lung progenitors at day 14 of alveolar organoid differentiation (Albumin: red, NKX2.1: green, DAPI: cyan). (E) Alveolar organoids at day 44 of differentiation (BU3NGST cell line; *NKX2.1*$^{GFP+}$, *SFTPC*$^{tdTomato+}$).

A

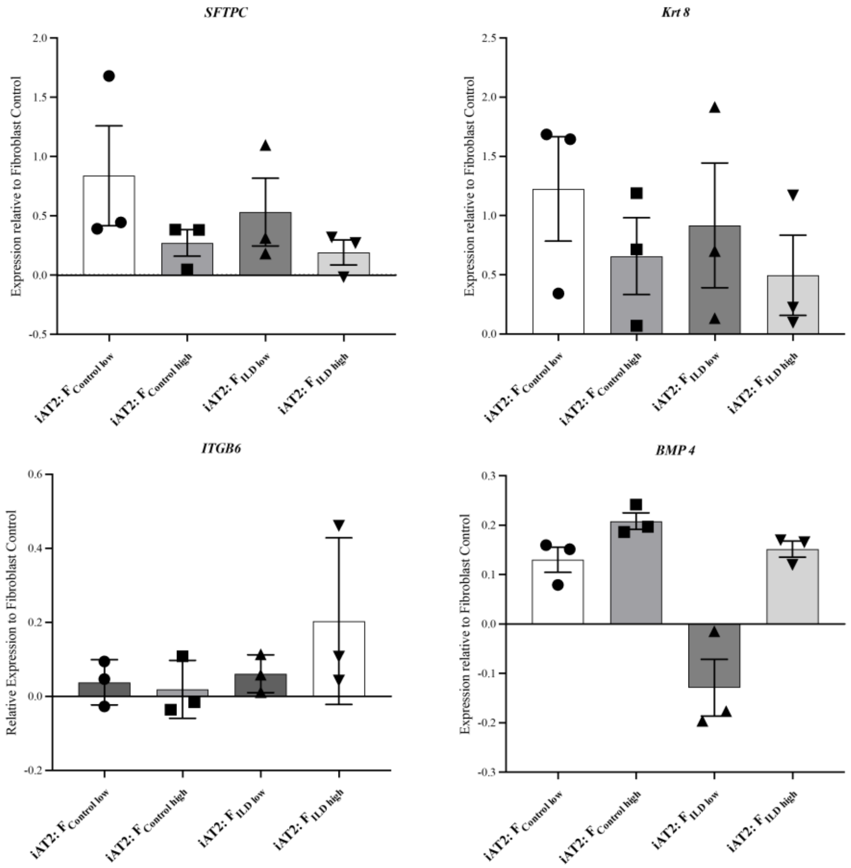

SFTPC
Krt 8
ITGB6
BMP 4
Expression relative to Fibroblast Control
Relative Expression to Fibroblast Control
iAT2: F Control low
iAT2: F Control high
iAT2: F ILD low
iAT2: F ILD high

B

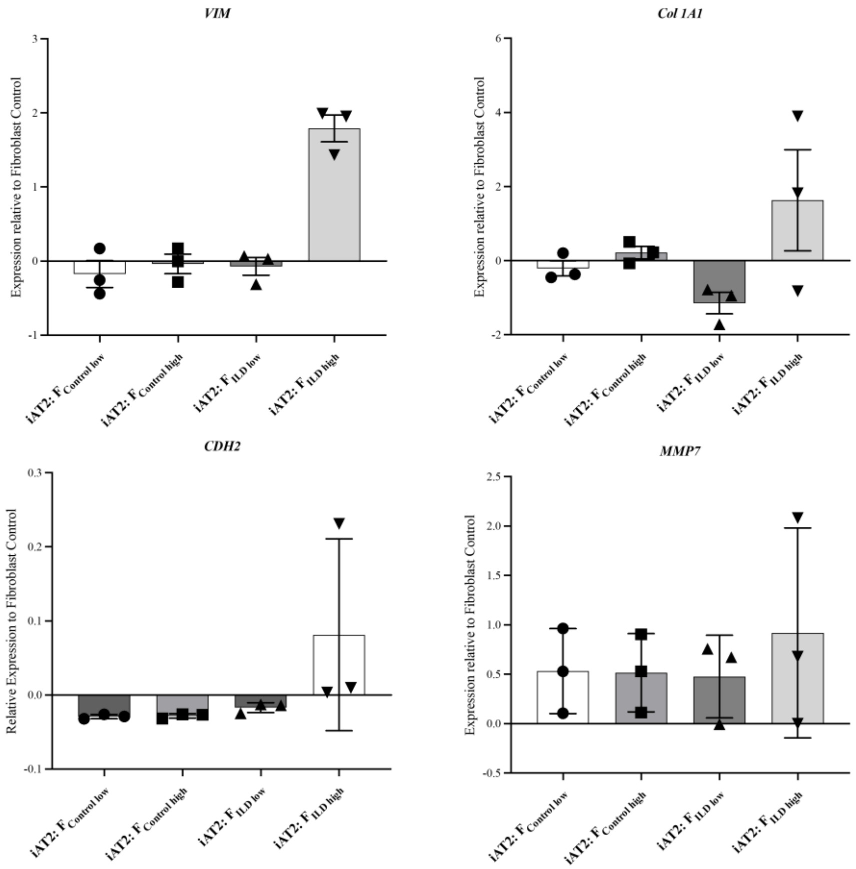

VIM
Col 1A1
CDH2
MMP7
Expression relative to Fibroblast Control
Relative Expression to Fibroblast Control
iAT2: F Control low
iAT2: F Control high
iAT2: F ILD low
iAT2: F ILD high

Supplementary Figure 2. Comparison of relative gene expression of co-cultures with mono-cultured ILD or IMR90 control fibroblasts to understand potential epithelial origin of observed gene expression changes in co-cultures. Co-cultures were obtained by culturing iAT2s with either IMR-90 control fibroblasts or ILD fibroblasts in two seeding ratios (F_{low} and F_{high}), N = 3. (A) Epithelial and stem cell markers and (B) genes associated with aberrant differentiation of epithelium.
